# The Knowledge of Contextual Factors as Triggers of Placebo and Nocebo Effects in Patients With Musculoskeletal Pain: Findings From a National Survey

**DOI:** 10.3389/fpsyt.2019.00478

**Published:** 2019-07-04

**Authors:** Giacomo Rossettini, Alvisa Palese, Tommaso Geri, Mattia Mirandola, Fabio Tortella, Marco Testa

**Affiliations:** ^1^Department of Neuroscience, Rehabilitation, Ophthalmology, Genetics, Maternal and Child Health, University of Genova, Savona, Italy; ^2^Department of Medical Sciences, University of Udine, Udine, Italy

**Keywords:** placebo effect, nocebo effect, pain, musculoskeletal, survey, conditioning, learning, expectation

## Abstract

**Backgrounds:** Contextual factors (CFs) have been recently proposed as triggers of placebo and nocebo effects in musculoskeletal pain. CFs encompass the features of the clinician (e.g. uniform), patient (e.g. expectations), patient–clinician relationship (e.g. verbal communication), treatment (e.g. overt therapy), and healthcare setting (e.g. design). To date, the researchers’ understanding of Italian patients’ knowledge about the role of CFs in musculoskeletal pain is lacking.

**Objectives:** The aim of this study was to investigate attitudes and beliefs of Italian patients with musculoskeletal pain about the use of CFs in clinical practice.

**Methods:** A national sample of Italian patients with musculoskeletal pain was recruited from 12 outpatient private clinics in Italy. An invitation to participate in an online survey was sent to patients: a) exhibiting musculoskeletal pain; b) aged 18–75; c) with a valid e-mail account; and d) understanding Italian language. Survey Monkey software was used to deliver the survey. The questionnaire was self-reported and included 17 questions and 2 clinical vignettes on the patients’ behavior, beliefs, and attitudes towards the adoption of CFs in clinical practice. Descriptive statistics and frequencies described the actual number of respondents to each question.

**Results:** One thousand one hundred twelve patients participated in the survey. Five hundred seventy-four participants were female (52%). The average age of patients was 41.7 ± 15.2 years. Patients defined CFs as an intervention with an unspecific effect (64.3%), but they believed in their clinical effectiveness. They identified several therapeutic effects of CFs for different health problems. Their use was considered ethically acceptable when it exerts beneficial psychological effects (60.4%), but it was banned if considered deceptive (51.1%). During clinical practice, patients wanted to be informed about the use of CFs (46.0%) that are accepted as an addition to other interventions to optimize clinical responses (39.3%). Moreover, patients explained the power of CFs through body–mind connections (37.1%).

**Conclusion:** Patients with musculoskeletal pain had positive attitudes towards the use and effectiveness of CFs when associated with evidence-based therapy. They mostly perceived the adoption of CFs in clinical practice as ethical.

## Introduction

Placebo and nocebo effects represent an emerging area of interest in musculoskeletal treatment. In this field, for several years, researchers have considered placebo and nocebo as incidental elements to be supervised in randomized controlled trials aimed at isolating the specific effect of a treatment (1). However, in the last decades, the modern neurobiological perspective has conceptualized placebo and nocebo effects as results of the psychosocial context surrounding every healthcare intervention, capable of influencing patients’ pain (2).

Placebo effects are the beneficial result of a patient’s exposure to a positive context (3), while nocebo effects are adverse consequences of a patient’s interaction with a negative context (4). Expectations and conditioning are the main psychological mechanisms underlying placebo and nocebo effects, although social learning and mindset theories have also been demonstrated as explanations of their existing and their functioning (5–7). From a neurobiological perspective, the release of specific neurotransmitters is associated with the exposure to specific contexts: endogenous opioids, dopamine, cannabinoids, oxytocin, and vasopressin have been observed in positive contexts, while opioid and dopamine deactivation and cholecystokinin and cyclooxygenase-prostaglandins activation were observed in negative contexts (8–11). Moreover, different contexts can modulate neural pathways involved in the descending control of pain, influencing the activity of anterior cingulate cortex, dorsolateral prefrontal cortex, periaqueductal grey, and spinal cord (12–16).

The context is composed of several therapeutic signs, symbols, metaphors, and healing rituals (17, 18), called Contextual Factors (CFs), that inform the patients on the value and the meaning of treatment delivered and can influence their healthcare experience by triggering placebo and nocebo effects (19). The therapeutic encounter is strongly characterized by CFs such as a) the clinicians’ beliefs and behaviors; b) the patients’ expectations and his/her previous experiences; c) the colour and the shape of the intervention; d) the verbal and non-verbal element of communication; and e) the ornaments and the colour of the healthcare setting (2). A robust body of evidence informs clinicians about the positive impact of CFs on therapeutic outcomes such as pain, disability, satisfaction, and perceived quality in different healthcare field as medicine, nursing, physiotherapy, musculoskeletal, and neurological rehabilitation (2, 20–23). As a consequence, a recent experts consensus suggested the adoption of CFs to stimulate placebo effects and to avoid nocebo effects, thus increasing the overall effectiveness of established evidence-based interventions (24).

From a clinical perspective, the patient’s point of view about CFs has been proposed as a central line of investigation (25). Up to now, qualitative and quantitative researches have investigated the participants’ point of view towards placebo using focus groups (26, 27), interviews (28–30), and surveys (31–39–44). Studies have been performed in different countries such as the US (28, 29, 32–33, 34, 38, 39), Asia (27, 42, 43), Australia (36), and Europe (26, 30, 31, 35, 37, 40, 41, 44), involving healthy subjects (26, 27, 32, 34, 41–43) and patients with acute/chronic health conditions (30, 36–39, 40, 44), depression (43), irritable bowel syndrome (28, 29), and rheumatic and musculoskeletal pain (31, 33, 35). Overall, findings revealed a) a heterogeneous understanding of placebo effects, ranging from limited (27, 32, 33, 35, 36, 40) to well-expressed knowledge (30, 31, 37, 39); b) a dualistic conceptualization of placebo effects, as a beneficial element to be legitimized or as ineffective (26, 28); and c) an open vision about placebos in clinical practice, revealing the deception and the lack of informed consent as major ethical issues of their use (27, 30, 32–38, 39, 44). However, the cultural differences and the various adopted definitions of “placebo treatment” threatened the development of a coherent body of evidence and require more research in the field (25, 39), particularly in Italy, where no studies have investigated the attitudes and beliefs of Italian patients towards CFs.

Moreover, among other different chronic conditions greatly affecting the quality of life of patients, musculoskeletal pain medicine represents an interesting and open field of investigation, given its high frequency and its pervasion by CFs (2). Aligned with this vision, the aims of our study were to explore: a) the clinical behaviors, b) the definition, c) the beliefs, d) the ethical concerning, e) the communication implications, f) the circumstances of application, and g) the mechanism of actions of CFs in a nationwide sample of Italian patients with musculoskeletal pain.

## Materials and Methods

### Design

A quantitative web-based cross-sectional survey herein reported in accordance with the Checklist for Reporting Results of Internet E-Surveys (CHERRIES) guidelines (45) and STrengthening the Reporting of OBservational Studies in Epidemiology (STROBE) (46) was performed. The Liguria Clinical Experimental Ethics Committee (P.R.236REG2016, accepted on 19/07/2016) approved the present study.

### Participants and Setting

A national sample of Italian patients with musculoskeletal pain was recruited from 12 outpatients’ private clinics located in different regions of Italy (North, *n* = 4; Centre, *n* = 4; South, *n* = 4) between May and August 2018.

Managers of each clinic provided the list of patients recruited for this survey to the principal investigator. The patients were included/excluded in accordance with the physician’s judgement based on the defined criteria. The inclusion criteria were as follows: a) age between 18 and 75 (38, 39); b) being currently affected by musculoskeletal pain due to either acute traumatic events (e.g., a fracture) or chronic complaints (e.g., overuse) (47); c) having a valid e-mail account; d) good understanding of the Italian language (33); and e) a EuroQol Index (EQI) < 1. The EQI has values ranging from 0 (worst) to 1 (best) and was calculated using the specific normative data of the Italian population (48). The EQI was calculated starting from the answers given in the EuroQol 5-dimensional scale (EQ-5D-3L), that is, a descriptive system composed of five closed three-level single answer questions, exploring mobility, self-care, usual activities, pain/discomfort, and anxiety/depression domains. Patients affected by cancer or by non-musculoskeletal cause of pain (e.g. neuropathic pain) (33) were excluded.

The number of eligible people who responded to the survey was 1,112. With this sample size, a relative standard error of 3% of the true estimate in the population with a 95% confidence level within 0.03 percentage points was expected, using a simple random sampling approach and with the population proportion set to 50% (49).

### Questionnaire Development and Pre-Testing

A survey instrument which included questions and clinical vignettes was developed adapting a previous survey on CFs performed among Italian physical therapists and nursing by our research group (50, 51). Questions and clinical vignettes were linguistically adapted to facilitate patient’s understanding and answers by the research group. In the whole questionnaire, the word “placebo” was avoided preferring the word “contextual factors” aimed at improving the number of responses by participants (26, 50–52).

The initial list was composed of 22 questions and 2 clinical vignettes that were critically appraised for face and content validity (53) using a panel of seven experts with a wide experience in placebo and survey design (a psychologist, a nurse, and five physical therapists). The experts checked the list independently providing feedback on content accuracy, relevance, wording clarity, and survey structure. Following the feedback received, some adjustments were made and the number of questions was reduced from 22 to 17 because there were overlapping and redundancy.

Once consensus on the final questionnaire was reached among the experts, a preliminary version of the survey, composed of 17 questions and 2 clinical vignettes, was piloted in a convenience sample of 45 patients with musculoskeletal pain and coming from different Italian regions (North, *n* = 15, Centre, *n* = 15; South, *n* = 15) (54).

After the pilot, a telephone debriefing session was performed (53). Experts interviewed the convenience sample of patients about the possible problems encountered during the survey (e.g. recognizing questions that needed additional explanation, wording that was hard to read or that participants found unclear). The outcome of the pilot phase offered the opportunity to reword three items (regarding ethics, communication, and mechanism of action) and to improve the readability of the entire survey.

### Questionnaire Implementation

The self-administered questionnaire (**Supplementary file 1** – English version, **Supplementary file 2** – Italian version) adopted in this study was divided into three sections (A, B, C), which used both open-ended and closed multiple-choice questions (55).

Section A investigated the socio-demographic variables using six questions (age, sex, geographical region, social status, workplace, and education). Three closed multiple-choice single answer questions explored the features of musculoskeletal pain (anatomical location, time of onset, and intensity using Numeric Rating Scale 0–10) (56).

In Section B, two clinical vignettes were presented as two closed multiple-choice questions with, respectively, single and multiple answers:

1. the first vignette was about the use of massage in a patient with low back pain and high expectations towards this treatment based on previous positive experience. Participant were asked to choose what they considered the best action in this situation in which the clinician knew that massage was not indicated and that the low back pain would have spontaneously disappeared in a short time;
2. the second vignette described a clinical case of patient with shoulder pain who responded positively when a sham laser (with power-off) replaced the active laser therapy. In this scenario, participants were asked to draw a conclusion about the efficacy and effectiveness of the sham laser.

Section C comprised eight closed questions. Three closed multiple-choice single answer questions investigated the definition of CFs (“How would you define the therapeutic role of CFs?”), the participants’ CFs belief (Likert from 0 “not at all” to 4 “a lot of”), and the potential beneficial effects of CFs (“What are the potential effects of CFs in the following health problems?”). Moreover, five closed multiple-choice multiple answers explored the ethical implications perceived in adopting CFs (e.g. “The use of CFs for therapeutic purposes can be considered ethically acceptable when….”), communication implications about CFs (“How do you communicate to the patient the use of CFs at the end of treatment?’), the circumstances under which they are applied (“Under what circumstances would you use CFs?”), and the possible mechanisms of action (“What mechanism of action can explain the effect of CFs?”).

### Data Collection Procedure

Survey Monkey (Survey-Monkey, Palo Alto, California, www.surveymonkey.com) online survey tool was adopted to administer the questionnaire. The survey was disseminated over a 12-week period between 18^th^ May 2018 and 18^th^ August 2018. Participants were contacted using the mailing list of the 12-outpatient private clinics (55). An email including the survey link (https://it.surveymonkey.com/r/contestopazientiitalianimsk) and a brief note outlining a) the aim of the study, b) data handling (anonymity), c) the informed consent statement, and d) the invitation to complete the survey was distributed. More specifically, the statement in the email informed the recipient that, by clicking on the survey link, the respondents were providing their consent to participate in the study (55). Moreover, an operational definition of CFs was provided to introduce participants to the topic, thus avoiding misinterpretation (30, 35–39): “CFs represent a series of relational or environmental situations capable of influencing the perception of your healthcare condition. Examples of CFs are: the words and posture used by the clinician, the smells, the sounds, and the furnishing of the therapeutic setting” (2).

Three email reminders were sent 4 and 8 and 12 weeks after the initial contact to encourage those who did not take part in the survey to complete it. The time required to complete the survey was 10–15 min (12 min on average), as per the optimal time required to increase response rates in online surveys (57). Participation was voluntary, and no incentives were offered to participants (55). Due to forced response validation, participants were required to answer all questions to prevent missing data (58). Participants were able to review or change responses using a back button before getting to the end of the questionnaire. At the end of the survey, a summary of the answers was provided to the participants (55). Data were copied and deposited in an encrypted computer, and only the project leader could access information achieved in all stages of the study (55). Participants’ identities remained concealed to researchers; all data were anonymized (names and mail addresses) to ensure confidentiality and data protection and to avoid psychological harm (55).

### Data Analysis

Survey data were downloaded from SurveyMonkey into .xls format and reviewed for data quality.

For descriptive statistics, continuous variables were reported using mean and standard deviation (SD). The five response options for the domain beliefs about CFs were also analyzed with mean and SD in order to have an average distribution of each single belief. Dichotomous, nominal, and ordinal variables, coming from single answer questions, were described using absolute and relative frequencies. Intervals of the observed estimates were calculated with a 95% confidence level (95%CI). For the questions with multiple answers, the absolute and relative frequencies were calculated for each combination of responses given by each participant. For example, considering that the fields (*n*) asked in the domain “Non-ethic” were three with dichotomous responses (*r*), we did not calculate the absolute frequency of the three possible fields, but of their eight combinations, given by the formula *r* ^∧^ *^n^*, to better describe the groups of participants giving multiple answers present in the population.

The association between the individual characteristics (section A of the survey) and the single choice responses given in sections B and C of the survey was investigated with Cramer’s V, which is a measure of strength and direction of association derived from chi-square statistics, which was not considered for the analysis of the differences because its significance depends on the size of the sample. For this purpose, age was transformed into ordinal variables considering a decade as variable levels for the analysis of correlations, as described below. Only correlation values above the threshold of acceptance set at 0.60 were reported.

Data analysis was handled using R software (59) and the psych (60) and ggplot2 (61) packages.

## Results

### Participants’ Characteristics

The majority of patients (*n* = 574; 51.6%; 95%CI 48.6–54.6) were female; their average age was 41.7 ± 15.2 years. 43.9% of participants (*n* = 488; 95%CI 40.9–46.9) were living in the North of Italy at the time of the survey.

Fifty point three percent of participants were high school graduate (*n* = 559; 95%CI 47.3–53.2); a large part of them were employed (*n* = 755; 67.9%; 95%CI 65.0–70.6) in intellectual, scientific, and highly specialized professions (*n* = 164; 14.7%; 95%CI 12.7–17.0).

Participants reported musculoskeletal pain principally located in the cervical spine and head region (*n* = 258; 23.2%; 95%CI 20.8–25.8). They had been suffering from pain for >6 months (*n* = 563; 50.6%; 95%CI 47.6–53.6) with a mean level of severity of 4.9 out of 10 (95%CI 4.8–5.0). The EQI presented a mean of 0.85 out of 1 ± 0.12.

The respondents’ demographics are described in **Table 1**.

**Table 1:** Participant characteristics (*n* = 1,112).

Demographic	Values	95%CI
Gender, *n* (%) Female Male	574 (51.6) 538 (48.4)	48.6–54.6 45.4–51.4
Years, mean (SD)	41.7 (15.2)	40.8–42.6
Italian region, *n* (%) North Centre South	488 (43.9) 323 (29) 301 (27.1)	40.9–46.9 26.4–31.8 24.5–29.8
Social status, *n* (%) Employed Student Retired Housewife Unemployed	755 (67.9) 149 (13.4) 119 (10.7) 59 (5.3) 30 (2.7)	65.0–70.6 11.5–15.6 9.0–12.7 4.1–6.8 1.9–3.9
Type of job*, *n* (%) Nothing Intellectual, scientific, highly specialized profession Trade, service Office workers Technician Laborer, farmer, artisan Unqualified profession Legislator, businessman, manager Drivers Military profession	357 (32.1) 164 (14.7) 162 (14.6) 139 (12.5) 96 (8.6) 94 (8.5) 36 (3.2) 25 (2.2) 24 (2.2) 15 (1.3)	29.4–34.9 12.7–17.0 12.6–16.8 10.6–14.6 7.1–10.5 6.9–10.3 2.3–4.5 1.5–3.3 1.4–3.2 0.8–2.3
Education, *n* (%) High school Bachelor’s degree Secondary school Master’s degree Primary school PhD	559 (50.3) 328 (29.5) 133 (12.0) 56 (5.0) 22 (1.9) 14 (1.3)	47.3–53.2 26.8–32.3 10.1–14.0 3.9–6.5 1.3–3.0 0.7–2.2
Anatomical region of pain, *n* (%) Cervical spine–head Lumbar spine–pelvis Shoulder–arm Knee–leg Ankle–foot Hip–thigh Thoracic spine–ribs Wrist–hand Elbow–forearm Jaw	258 (23.2) 252 (22.7) 193 (17.4) 155 (13.9) 70 (6.3) 55 (4.9) 47 (4.2) 44 (4.0) 25 (2.2) 13 (1.2)	20.8–25.8 20.2–25.3 15.2–19.7 12.0–16.1 5.0–7.9 3.8–6.4 3.1–5.6 2.9–5.3 1.5–3.3 0.6–2.0
Duration of pain, *n* (%) Over 6 months Less than 3 months From 3 to 6 months	563 (50.6) 355 (31.9) 194 (17.4)	47.6–53.6 29.2–34.8 15.3–19.8
Intensity of pain, mean (SD)	4.9 (2.1)	4.8–5.0
EQI, mean (SD)	0.85 (0.12)	0.85–0.86

n, number of participants; %, percentage; SD, standard deviation; 95%CI, 95% confidence interval; >, more; visual analog scale, visual; EQI, EuroQol Index.

*According to “Nomenclature and classification of work” provided by ISTAT http://professioni.istat.it/sistemainformativoprofessioni/cp2011/

### Clinical Vignette 1

**Figure 1:**
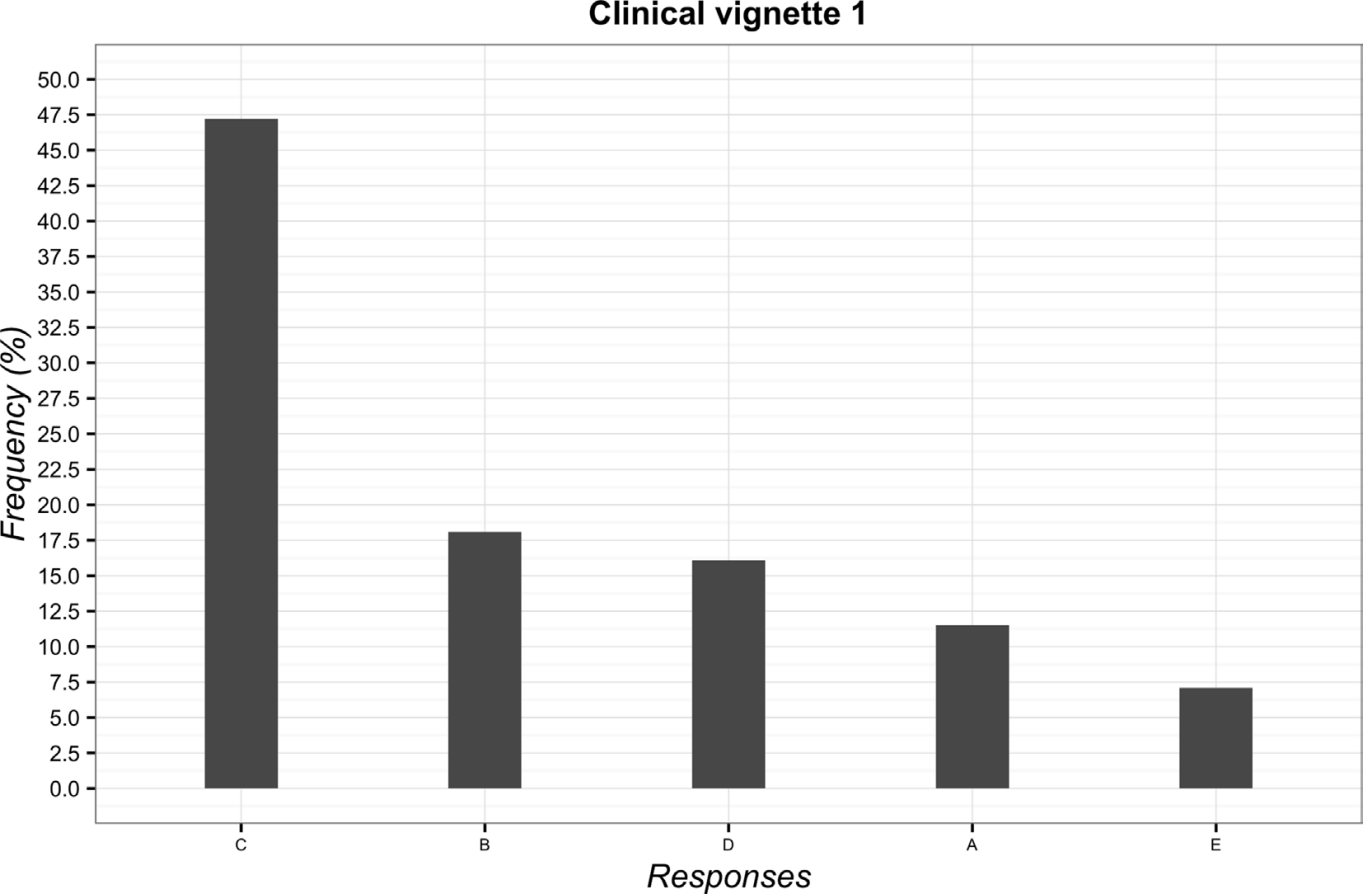
Percentages of responses for clinical vignette 1. **(A)** deliver massage; **(B)** tell the patient that low back pain would resolve itself in a few days; **(C)** suggest the possibility of delivering massage if the clinical condition fails to improve; **(D)** advise a different treatment commonly used for low back pain; **(E)** try to convince the patient of the futility of the massage.

The most frequently chosen solution to the first vignette was “to suggest the possibility of delivering massage if the clinical condition fails to improve” (*n* = 525; 47.2%; 95%CI 44.2–50.2). The least frequent answer instead was to “try to convince the patient of the uselessness of massage” (*n* = 79; 7.1%; 95%CI 5.7–8.8). The overall overview of data is reported in **Figure 1**.

### Clinical Vignette 2

**Figure 2:**
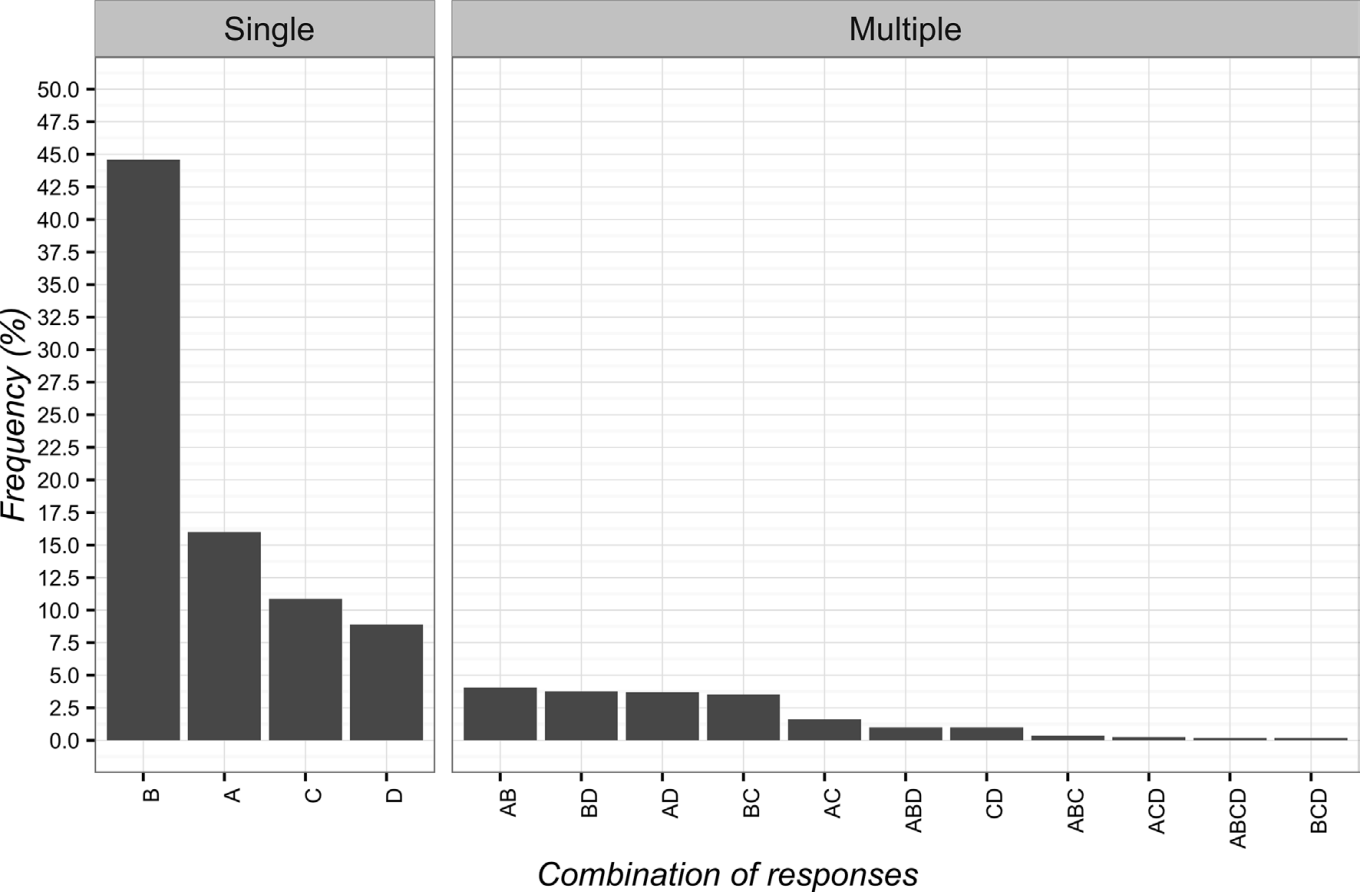
Percentages of responses for clinical vignette 2. **(A)** the positive attention of the healthcare team leads to decreased pain; **(B)** pain is not organic but psychological; **(C)** the patient is very suggestible; **(D)** the supporting patient saw an improvement after treatment with laser switched off.

The most frequent answer to the second vignette was “pain is not organic but psychological” (*n* = 496; 44.6%; 95%CI 41.7–47.6), while the least frequent one was “supporting patient determined improvements after treatment with sham laser (power-off)” (*n* = 99; 8.9%; 95%CI 7.3–10.8). The single items and their combinations are presented in **Figure 2**.

### Definition of CFs

The majority of patients defined CFs as “an intervention without a specific effect for the condition being treated, but with a possible unspecific effect” (*n* = 715; 64.3%; 95%CI 61.4–67.1). Instead, the minority of patients identified CFs as “a sham treatment used as control tests for safety and efficacy of active treatment” (*n* = 109; 9.8%; 95%CI 8.1–11.7). The remaining considered CFs as “a harmless or inert intervention” (*n* = 167; 15.0%; 95%CI 13.0–17.3) or “an intervention that has a special effect through known physiological mechanisms” (*n* = 121; 10.9%; 95%CI 9.1–12.9).

### Beliefs

The mean score of beliefs was 2.6 out of 5 (95%CI 2.5–2.6), thus denoting a substantial level of belief towards CFs among patients. In detail, the most believed CFs were (in descending order): “overt therapy” (mean = 3.4; 95%CI 3.3–3.4), “empathetic therapeutic alliance with the patient” (mean = 3.3; 95%CI 3.2–3.3), “verbal communication” (mean = 3.1; 95%CI 3.0–3.1), and “patient-centered approach” (mean = 3.1; 95%CI 3.0–3.1). The least believed CFs were (in descending order): “adequate design” (mean = 1.8; 95%CI 1.8–1.9), “uniform” (mean = 1.8; 95%CI 1.8–1.9), and “physical contact with the patient” (mean = 1.5; 95%CI 1.4–1.5). An overall description of beliefs towards CFs is presented in **Table 2**.

**Table 2:** Beliefs regarding contextual factors (*n* = 1,112).

Contextual factor items^a^	Likert score mean (95%CI)	4 *n* (%); 95%CI	3 *n* (%); 95%CI	2 *n* (%); 95%CI	1 *n* (%); 95%CI	0 *n* (%); 95%CI
**A: Professional reputation** (e.g. qualification, expertise)	2.1 (2.0–2.1)	70 (6.3); 5.0–7.9	266 (23.9); 21.5– 26.6	513 (46.1); 43.2–49.1	192 (17.3); 15.1–19.6	71 (6.4); 5.0–8.0
**A: Uniform** (e.g. white coat)	1.8 (1.8–1.9)	65 (5.8); 4.6–7.4	229 (20.6) 18.3–23.1	431 (38.8); 35.9–41.7	241 (21.7); 19.3–24.2	146 (13.1); 11.2–15.3
**A: Positive attitudes and behavior** (e.g. towards a patient’s dysfunctions)	2.7 (2.6–2.7)	156 (14.0) 12.1–16.2	577 (51.9) 48.9–54.9	276 (24.8); 22.3–27.5	78 (7.0); 5.6–8.7	25 (2.2); 1.5–3.4
**B: Patient’s expectation and preference** (e.g. towards a treatment)	2.2 (2.2–2.3)	97 (8.7); 7.2–10.6	408 (36.7); 33.9–39.6	326 (29.3); 26.7–32.1	210 (18.9); 16.7–21.3	71 (6.4); 5.1–8.0
**B: Patient’s previous experience** (e.g. towards a treatment)	2.7 (2.7–2.8)	168 (15.1); 13.1–17.4	586 (52.7); 49.7–55.7	272 (24.5); 22.0–27.1	64 (5.8); 4.5–7.3	22 (2.0); 1.3–3.0
**C: Verbal communication** (e.g. positive messages associated with the treatment)	3.1 (3.0–3.1)	334 (30.0); 27.4–32.8	582 (52.3); 49.3–55.3	151 (13.6); 11.6–15.8	29 (2.6); 1.8–3.8	16 (1.4); 0.9–2.4
**C: Non-verbal communication** (e.g. posture, gestures, eye contact, facial expressions)	2.9 (2.8–2.9)	251 (22.6); 20.2–25.2	572 (51.4); 48.5–54.4	205 (18.4); 16.2–20.9	62 (5.6); 4.3–7.1	22 (2.0); 1.3–3.0
**C: Empathetic therapeutic alliance with the patient** (e.g. active listening)	3.3 (3.2–3.3)	545 (49.0); 46.0–52.0	384 (34.6); 31.8–37.4	136 (12.2); 10.4–14.3	40 (3.6); 2.6–4.9	7 (0.6); 0.3–1.4
**D: Overt therapy** (e.g. possibility for the patient to see the treatment using a mirror)	3.4 (3.3–3.4)	578 (52.0); 49.0–54.9	398 (35.8); 33.0–38.7	99 (8.9); 7.3–10.8	29 (2.6); 1.8–3.8	8 (0.7); 0.3–1.5
**D: Patient-centered approach** (e.g. shared-decision of treatment)	3.1 (3.0–3.1)	346 (31.1); 28.4–33.9	578 (52.0); 49.0–54.9	150 (13.5); 11.6–15.7	26 (2.3); 1.6–3.5	12 (1.1); 0.6–1.9
**D: Professional approach to patient** (e.g. privacy, punctuality)	3.0 (3.0–3.1)	380 (34.2) 31.4–37.1	496 (44.6); 41.6–47.6	154 (13.8); 11.9–16.1	56 (5.0); 3.9–6.5	26 (2.3); 1.6–3.5
**D: Physical contact with the patient** (e.g. touch to inform, assist, prepare, take care)	1.5 (1.4–1.5)	63 (5.7); 4.4–7.2	168 (5.7); 13.1–17.4	200 (18.0); 15.8–20.4	491 (44.2); 41.2–47.1	190 (17.1); 14.9–19.5
**E: Comfortable setting** (e.g. little noise, music, fragrances, temperature)	2.7 (2.6–2.7)	174 (15.6); 13.6–17.9	562 (50.5); 47.6–53.5	259 (23.3); 20.9–25.9	86 (7.7); 6.3–9.5	31 (2.8); 1.9–4.0
**E: Adequate environmental architecture** (e.g. windows and skylights, supportive indicators)	2.3 (2.2–2.4)	120 (10.8); 9.1–12.8	338 (30.4); 27.7–33.2	456 (41.0); 38.1–44.0	151 (13.6); 11.6–15.8	47 (4.2); 3.2–5.6
**E: Adequate design** (e.g. decorations, ornaments and colors)	1.8 (1.8–1.9)	63 (5.7); 4.4–7.2	189 (17.0); 14.9–19.4	478 (43.0); 40.1–46.0	286 (25.7); 23.2–28.4	96 (8.6); 7.1–10.5

%, percentage; n, number of participants; 95%CI, 95% confidence interval; 0, not at all; 1, few; 2, enough; 3, much; 4, a lot of; A, physical therapist domain; B, patient domain; C, physical therapist–patient relationship domain; D, therapy domain; E, healthcare setting domain.

a The items were reported from: Testa M, Rossettini G. Enhance placebo, avoid nocebo: How contextual factors affect physiotherapy outcomes. Man Ther. 2016;24:65–74.

### Therapeutic Effect

Patients mainly chose “physiological and psychological” therapeutic effects for health problems such as acute pain (*n* = 640; 57.5%; 95%CI 54.6–60.5), chronic pain (*n* = 629; 56.6%; 95%CI 53.6–59.5), and insomnia (*n* = 562; 50.5%; 95%CI 47.6–53.5). The “psychological” effect was predominantly reported for emotional (*n* = 689; 62.0%; 95%CI 59.0–64.8) and cognitive disorders (*n* = 616; 55.4%; 95%CI 52.4–58.3) and oncological problems (*n* = 513; 46.1%; 95%CI 43.2–49.1). Patients identified the therapeutic effects behind several health conditions such as gastrointestinal (*n* = 451; 40.6%; 95%CI 37.7–43.5) and cardiovascular problems (*n* = 405; 36.4%; 95%CI 33.6–39.3) as “physiological.” Infectious (*n* = 629; 56.6%; 95%CI 53.6–59.5), immune/allergic (*n* = 566; 50.9%; 95%CI 47.9–53.9), drug, and medication addictions (*n* = 531; 47.8%; 95%CI 44.8–50.7) were selected as having “no benefit.” An overall report of therapeutic effects is presented in **Table 3**.

**Table 3:** Therapeutic effect(s) of contextual factors (*n* = 1,112).

Clinical conditions	Psychological and physiological *n* (%); 95%CI	Psychological *n* (%); 95%CI	Physiological *n* (%); 95%CI	No benefit *n* (%); 95%CI
Acute pain	640 (57.5); 54.6–60.5	210 (18.9); 16.6–21.3	132 (11.9); 10.1–13.9	130 (11.7); 9.9–13.8
Chronic pain	629 (56.6); 53.6–59.5	244 (21.9); 19.6–24.5	123 (11.1); 9.3–13.1	116 (10.4); 8.7–12.4
Cognitive disorder	227 (20.4); 18.1–22.9	616 (55.4); 52.4–58.3	65 (5.8); 4.6–7.4	204 (18.3); 16.1–20.8
Emotional disorder	336 (30.2); 27.5–33.0	689 (62.0); 59.0–64.8	50 (4.5); 3.4–5.9	37 (3.3); 2.4–4.6
Gastrointestinal problem	367 (33.0); 30.3–35.9	134 (12.0); 10.2–14.1	451 (40.6); 37.7–43.5	160 (14.4); 12.4–16.6
Sexual problem	505 (45.4); 42.5–48.4	336 (30.2); 27.5–33.0	99 (8.9); 7.3–10.8	172 (15.5); 13.4–17.8
Drug and medication addiction	283 (25.4); 22.9–28.1	258 (23.2); 20.8–25.8	40 (3.6); 2.6–4.9	531 (47.8); 44.8–50.7
Neurological problem	471 (42.4); 39.4–45.3	244 (21.9); 19.6–24.5	198 (17.8); 15.6–20.2	199 (17.9); 15.7–20.3
Rheumatologic problem	452 (40.6); 37.7–43.6	251 (22.6); 20.2–25.2	257 (23.1); 20.7–25.7	152 (13.7); 11.7–15.9
Immune/allergic problem	227 (20.4); 18.1–22.9	150 (13.5); 11.6–15.7	169 (15.2); 13.2–17.5	566 (50.9); 47.9–53.9
Oncological problem	310 (27.9); 25.3–30.6	513 (46.1); 43.2–49.1	74 (6.6); 5.3–8.3	215 (19.3); 17.1–21.8
Cardiovascular problem	297 (26.7); 24.1–29.4	185 (16.6); 14.5–19.0	405 (36.4); 33.6–39.3	225 (20.2); 17.9–22.7
Infectious problem	191 (17.2); 15.0–19.5	125 (11.2); 9.5–13.3	167 (15.0); 13.0–17.3	629 (56.6); 53.6–59.5
Insomnia	562 (50.5); 47.6–53.5	413 (37.1); 34.3–40.1	50 (4.5); 3.4–5.9	87 (7.8); 6.3–9.6

%, percentage; n, number of participants; 95%CI, 95% confidence interval.

### Ethical Implications

**Figure 3:**
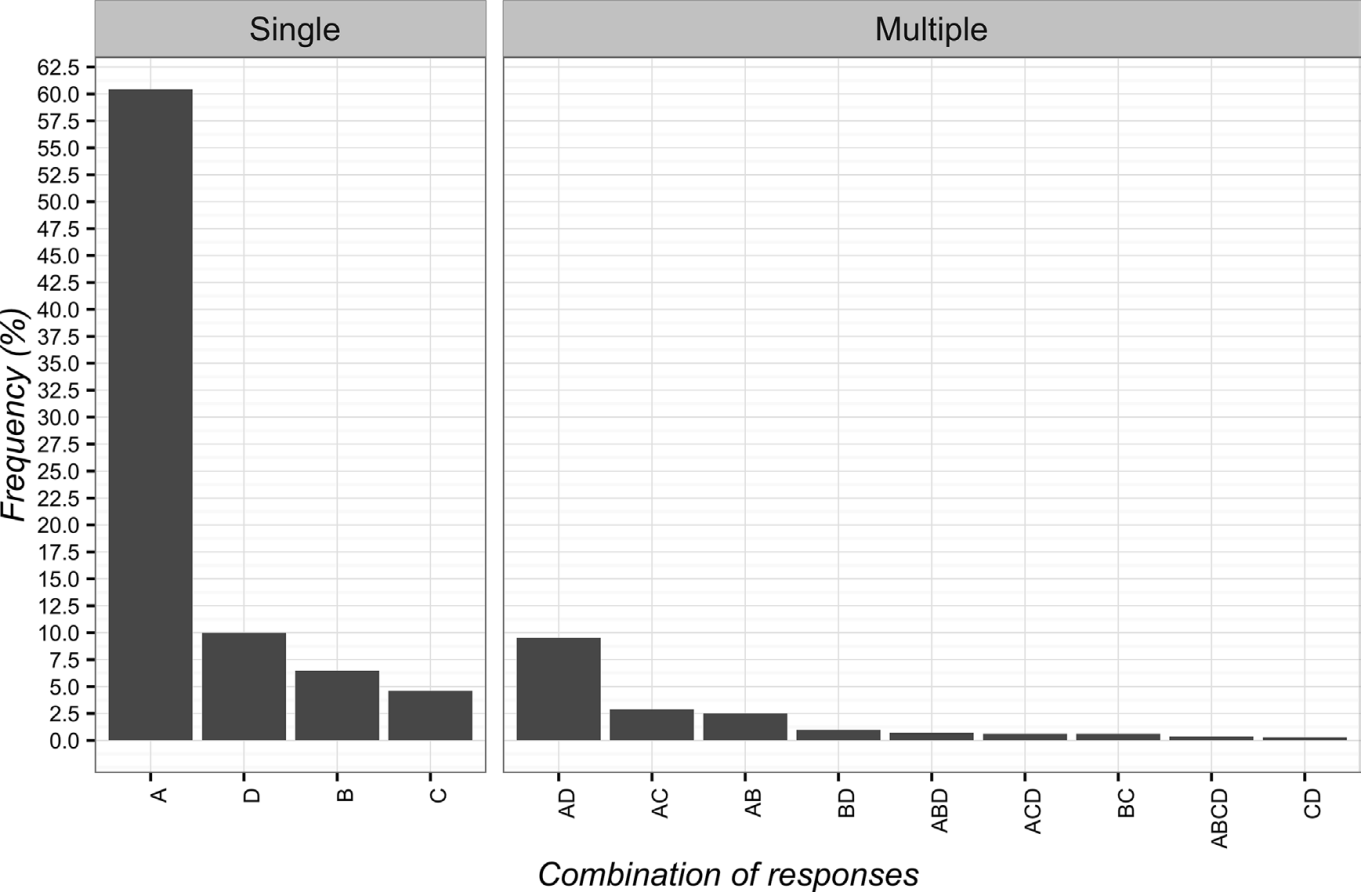
Percentages of responses for the ethical use of Contextual Factors. **(A)** it exerts beneficial psychological effects; **(B)** the other therapies are over; **(C)** the patient wants or expects this treatment; **(D)** effectiveness shown by clinical experience.

The adoption of CFs was considered ethical when “it exerts beneficial psychological effects” (*n* = 672; 60.4%; 95%CI 57.5–63.3). In this field, the least selected answer was “the patient wants or expects this treatment” (*n* = 51; 4.6%; 95%CI 3.5–6.0). The detailed responses are presented in **Figure 3**.

**Figure 4:**
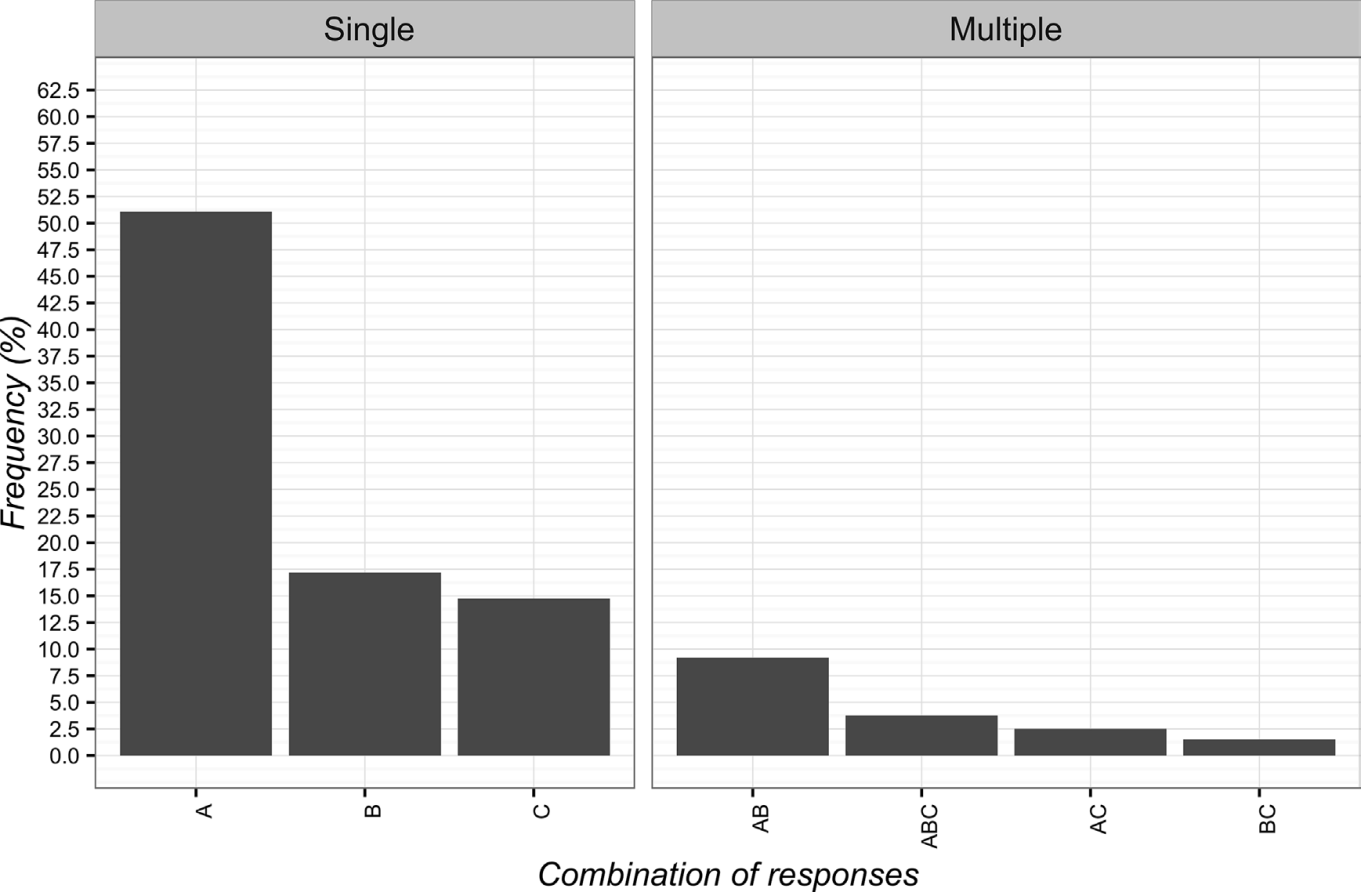
Percentages of responses for not-ethical use of Contextual Factors. **(A)** it is based on deception; **(B)** it undermines trust between patient and clinician; **(C)** the evidence is insufficient.

The adoption of CFs was instead considered non-ethical when “it is based on deception” (*n* = 568; 51.1%; 95%CI 48.1–54.0). Differently, the least frequent selected answer was when “the evidence available is insufficient” (*n* = 164; 14.7%; 95%CI 12.7–17.0). The overall responses are presented in **Figure 4**.

### Communication

**Figure 5:**
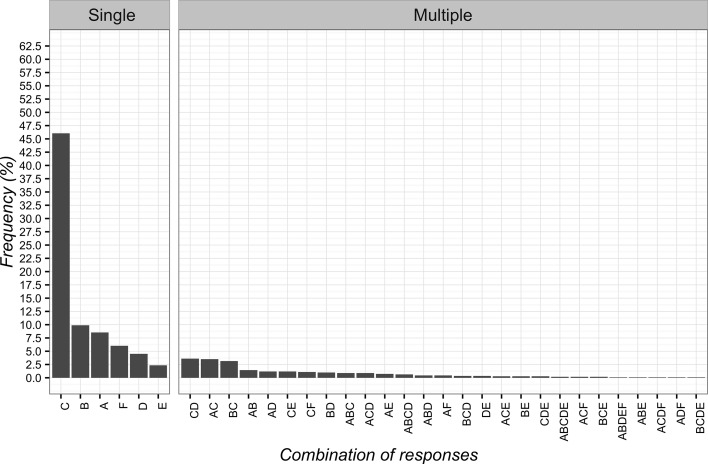
Percentages of responses for communicating to patients the implications of Contextual Factors. **(A)** it is a treatment that can help and will not hurt; **(B)** it is an effective treatment; **(C)** it is a treatment without a specific effect for your problem, but capable of improving your condition; **(D)** it is a treatment that induces a psychological change; **(E)** it can help but you are not sure about its effect; **(F)** you do not receive any information.

Participants desired to be informed about the use of CFs, thus selecting with a higher frequency the communication “it is a treatment without a specific effect for your problem, but capable of improving your condition” (*n* = 512; 46.0%; 95%CI 43.1–49.0). The least frequent chosen item was “it can help but you are not sure about its effect” (*n* = 26; 2.3%; 95%CI 1.6–3.5). The full combinations of responses are reported in **Figure 5**.

### Circumstances of CF Application and Mechanism of Action

**Figure 6:**
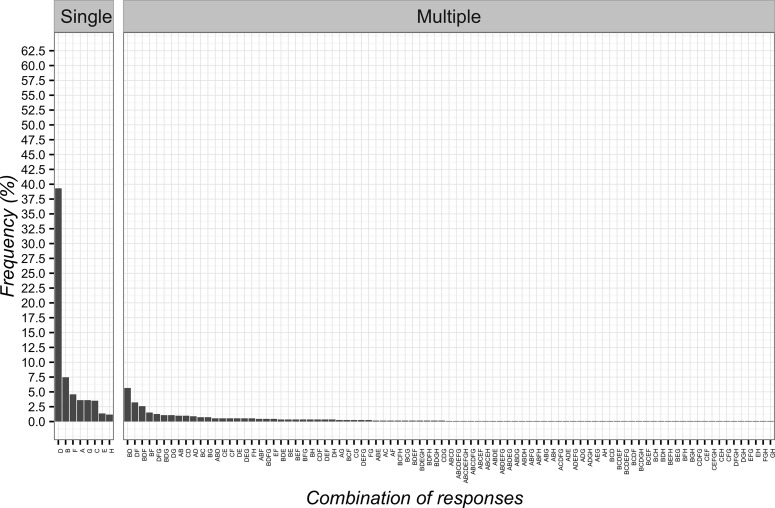
Percentages of responses for circumstances of Contextual Factors application. **(A)** as a result of unjustified and constant demands for healthcare interventions; **(B)** to calm down the patient; **(C)** when all other therapies are over; **(D)** in addition to other interventions to optimize clinical responses; **(E)** for non-specific problems; **(F)** to gain time; **(G)** as a diagnostic tool to differentiate between psychological and physiological problems; **(H)** to control pain.

As for the circumstances of CF application, the most frequent item was “as an adjunct to other interventions to optimize clinical responses” (*n* = 437; 39.3%; 95%CI 36.4–42.2). The least frequent answers were two items: “for non-specific problems” (*n* = 15; 1.3%; 95%CI 0.8–2.3) and “to control pain” (*n* = 13; 1.2%; 95%CI 0.6–2.0). Globally, the combinations of responses are presented in **Figure 6**.

**Figure 7:**
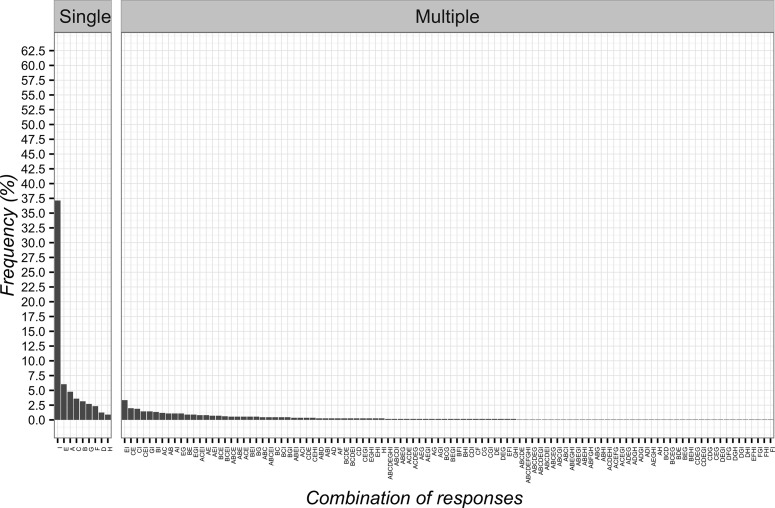
Percentages of responses for Contextual Factors mechanism of action. **(A)** patient’s expectation; **(B)** conditioning; **(C)** suggestibility; **(D)** natural history of disease; **(E)** psychological factors; **(F)** unexplained; **(G)** physiological/biological factors; **(H)** spiritual energies; **(I)** mind–body connections.

In terms of mechanism of action, patients selected “mind–body connections” as most frequent option (*n* = 413; 37.1%; 95%CI 34.3–40.1). The least frequent answers were instead “natural history of disease” (*n* = 14; 1.3%; 95%CI 0.7–2.2) and “spiritual energies” (*n* = 10; 0.9%; 95%CI 0.5–1.7) as reported in **Figure 7**.

### Correlation between Variables

The strength of association was considered weak with a Cramer’s V lower than the established threshold (Cramer’s V < 0.60) for all the correlations, such as between the characteristics reported in **Table 1** (gender, age, Italian region, social status, type of job, education, anatomical region of pain, duration of pain, intensity of pain, EQI) and the responses given in sections B and C of the survey.

## Discussion

To the best of our knowledge, this is the first research investigating the awareness of Italian patients about the therapeutic effect of CFs on musculoskeletal pain. The main findings of our study suggest that patients: a) conceptualized CFs as an intervention with an unspecific effect; b) believed in the clinical effectiveness of CFs; c) identified several possible therapeutic effects of CFs for various health problems; d) considered the use of CFs to stimulate beneficial psychological effects as ethically correct; e) saw as non-ethical the deceptive adoption of CFs; f) desired transparent information about CFs; g) recognized the application CFs as an adjunct to other interventions to optimize clinical responses; and h) proposed mind–body connection as a principal mechanism of action of CFs.

Therefore, according to our and former findings, it is recommended to extend the consideration of CFs in clinical policies and research designs, as they are also a patients’ perspective expression, and not only a significant contribution to the therapeutic outcome from clinicians’ point of view (18, 21, 25, 26, 29, 34). Namely, if patients present an adequate knowledge of CFs, their implementation can be ethically acceptable by clinicians and researchers. On the contrary, if patients report a misconception about CFs, clinicians and researchers should adequately reconceptualise their point of view before adopting CFs.

Responding to clinical vignette 1, about 50% of our participants suggested the possibility of delivering the expected intervention (massage) if clinical condition did not improve. As reported in previous qualitative researches (62, 63), patients with low back pain considered the fulfilment of expectation as a milestone of the decision-making process capable of improving clinical outcome(s) and adherence to treatment; therefore, clinicians should adopt it aimed at enhancing therapeutic responses (2). However former studies did not explore the ethical implications we proposed to patients in our survey. Our observations made clinicians aware that satisfying patients’ expectations cannot exceed ethical boundaries of professional deontology not only for their personal moral values but also for specific willingness of the patients. In other words, they desire that clinician avoids the administration of the expected intervention when it is detrimental or simply useless.

As resulted in clinical vignette 2, the majority of Italian patients considered the recovery of shoulder pain with laser switched off as explained by symptoms of psychological origin. In accordance with previous international surveys on placebo (30, 35, 36), participants recognized the patients’ psychological profile as an important predictor of placebo effects, able to explain the reduction in complaints (64). Therefore, clinicians should remember that patients are aware that their psychological condition affects their health status, so healthcare providers may have to weight this component in each healthcare interaction they have.

Our results made us consider CFs as an intervention lacking specificity capable of influencing patients’ clinical condition through an unspecific effect. This confirms the patients’ vision of placebo as an inert (32, 39), sham (37), fake (28) substance without any pharmacological active ingredient (30) rather than an active contextual process (25). This old conceptualization of placebos among patients can be the result of the patients’ socio-cultural context (education, friends and family) (29) and of the external information received (books, newspapers, social media, and the internet) (26, 52). Routinely, clinicians should assess their patients’ knowledge on placebo effects and try to correct misconceptions and inconsistencies with the current scientific thinking (65), for example, by encouraging the acquisition of information from evidence-based websites (66).

In line with previous surveys on placebos (30, 31, 35–39), Italian patients believed that CFs can influence therapeutic outcome(s). Namely, the most believed CFs are related to the therapeutic encounter (e.g. empathetic therapeutic alliance, communication, and overt therapy); the least believed CFs concerned healthcare design, the clinician’s uniform and the touch. Previous surveys focused on evaluating patients’ given value only on a part of possible CFs in each study, never trying to draft an importance ranking (26, 28, 31, 35, 37, 39). In our study, we aimed to draw up a classification, but this result suggests that patients assign the therapeutic value of CFs on a case-by-case basis. From a translational perspective, this finding pushes clinicians to assess patients’ beliefs about specific CFs in order to adopt and reinforce the CFs most believed to trigger placebo and to reduce nocebo effects.

Italian patients identified several therapeutic effects of CFs for various health problems ranging from physiological and psychological issues to no benefit. While in previous surveys the expected therapeutic effect was limited to diseases in which psychological influence plays an important role (pain) (30, 31, 35, 36, 39), our participants’ responses seem to be more articulated and support the idea that: a) CFs do not work in all diseases; b) CFs can act with different therapeutic effects (e.g. physiological and psychological); and c) the therapeutic effect of CFs depends on the specific nature and the severity of the disease. This heterogeneity could be related to the ethno cultural background that differ between patients from Northern (e.g. United Kingdom) and Southern Europe (e.g. Italy), and between European patients compared to other populations from different continents, as reported in former surveys on placebo (67). However, our findings are not conclusive, requiring further studies aimed at identifying patients’ perspective on the therapeutic effects of CFs in different health problems.

In accordance with the position of a recent expert consensus on placebo and nocebo for clinical practice (24), the majority of Italian patients considered as ethical and acceptable the use of CFs as therapy enhancers when they stimulate beneficial psychological effects and improve patients’ symptoms. The pursuit of patients’ benefit, the lack of harm, the absence of other effective treatments, and the presence of pain or other conditions of suffering are other main reasons for the ethical implementation of placebo treatments reported in literature (26, 30, 32–33, 34, 36–40). On the contrary, among surveys, the use of placebo is considered as non-ethical when: a) it conflicts with available scientific evidence; b) it provides advantages to clinicians; c) it determines dysfunctional attachment behavior between clinicians and patients; d) it is harmful; or e) it worsens clinical outcomes (26, 32–33, 34, 36, 38, 40).

Our participants considered as non-ethical the deceptive use of CFs. In accordance with previous surveys on placebo (26, 33, 34, 38, 44), deception was considered negatively as it determines a violation of the patients’ autonomy and right to be informed about the treatment delivered. Indeed, it can compromise the trust towards clinicians particularly when deceptive treatment resulted in negative outcomes (37, 39). Surprisingly, in other surveys, participants expressed a more tolerant opinion and considered deception acceptable when it helps patients to improve without damaging patient–clinician relationship (36, 41–43). The heterogeneity of these data highlights the complexity behind the ethical domain of CFs, thus the need for further research on the topic across countries.

As for communication, the majority of Italian patients desired transparent information about CFs. In line with previous surveys (26, 30, 37–38, 39, 44), our result confirms the need to notify patients without lying when they receive a non-specific treatment. Communication is a central aspect of the patient–clinician relationship and constitutes one of the most important CFs capable of triggering placebo or nocebo response with a relevant effect on clinical outcomes (2). Two strategies to inform patients have been reported in literature: 1) a direct message (“this is a placebo pill”) (37–39) or 2) an indirect general message (“this pill has helped others in the past”) that avoids the “placebo” word to limit misunderstanding related to the term (26, 30). Nevertheless, some results of previous surveys supported the non-transparent use of placebo treatments (35, 38–40): some respondents claimed that a clinician should not tell patients that the treatment was a placebo to avoid a potential lack of benefit. Currently, this vision appears dated and incompatible with the evidence available on several health conditions such as irritable bowel syndrome, depression, allergic rhinitis, back pain, and attention deficit hyperactivity disorder (68) that report positive clinical effects also to open-label placebo administration.

According to a previous survey among patients with musculoskeletal complaints (33), in our investigation, CFs are mainly seen by Italian patients as additional interventions that can optimize clinical responses. Overall, our finding suggests a patient’s positive attitude towards CFs, thus stimulating their adoption among clinicians to boost the result of evidence-based interventions (2, 22).

Mind–body connection has been proposed as the main mechanism of action of CFs by participants, in accordance with previous surveys on placebo (26, 28, 30, 38). Within a Cartesian dualistic perspective, the power of mind is able to activate patients’ inner resources and capacity of self-healing, thus directly influencing symptoms from body (28), relegating to a less relevant role other mechanisms such as expectation, conditioning, hope, psychological (e.g. attitudes, beliefs, and desire), and physiological factors (e.g. real change in the brain) (26, 28, 29, 31, 69, 70). The future analysis about the mechanisms behind the clinical effectiveness of CFs represents a research agenda capable to enrich the knowledge of patients’ perspective involved in the creation of placebo/nocebo effects.

## Strengths and Limitations

We have investigated for the first time the knowledge of CFs among Italian patients with musculoskeletal pain, thus expanding, also by involving a wider sample, findings of research in this field previously conducted in other countries (31, 33). Furthermore, the patients’ health status as measured with the EQI was similar to that of the general population (48). Compared to focus group methodology, the use of a questionnaire-based survey has contributed to expand the focus of our analysis and revealed the complexity behind CF construct (71). Moreover, the adoption of clinical vignettes helped to gradually introduce a potentially unfamiliar topic such as CFs to patients (26).

Despite the novelty of this study, we recognize several limitations that could affect our findings. First, we have recruited only participants from outpatient clinics, thus limiting the generalization of findings in different contexts (e.g. inpatient services). Second, although not correlated to CF knowledge in our sample, participants had a generally high education and work position, introducing a possible source of bias (38). Third, social desirability and recall bias could have occurred due to self-reported and retrospective nature of data (36, 37). Finally, the distribution of response in question with multiple choice (either with single or multiple answers) revealed the presence of different strata. Therefore, the confidence level of the estimate varies when the proportion of responses is different from the estimated 50% that occurred in non-dichotomic questions. We suggest using our result in future research to estimate the required sample size more precisely using stratified random sampling.

## Conclusion

Italian outpatient with musculoskeletal pain reported positive attitudes and beliefs towards the implementation of CFs in clinical practice, and this may have an impact at different levels.

According to the patients’ opinion, it is ethically welcome for clinicians to adopt CFs as an additional treatment integrated with the evidence-based intervention aimed at enhancing therapeutic outcomes.

To support a mindful clinical use of CFs, educational courses should be implemented in academic curricula to expand the knowledge among healthcare providers.

Moreover, following the patients’ vision, policymakers and managers should create the conditions and the normative frame to ease the appropriate integration of CFs in clinical practice.

Future surveys are needed to explore how patients conceptualise mechanisms of actions and the role of CFs in different health conditions and across countries.

## Ethics Statement

The present study consists in a web-based survey of which protocol was approved by the Liguria Clinical Experimental Ethics Committee (P.R.236REG2016, accepted on 19/07/2016). Participants were contacted by an email including the survey link (https://it.surveymonkey.com/r/contestopazientiitalianimsk) and a brief note outlining a) the aim of the study, b) data handling (anonymity), c) the informed consent statement, and d) the invitation to complete the survey. Moreover, the email specifically informed the recipient that by clicking on the survey link he would have provided his consent to participate in the study. Participants’ identities remained concealed to researchers; all data were anonymized (name and email address) to ensure confidentiality and data protection.

## Author Contributions

Conceptualization: GR, MT. Data curation: GR, TG. Formal analysis: TG. Investigation: GR, MT. Methodology: GR, AP, TG, MM, FT, MT. Project administration: GR, MT. Resources: GR, MT. Software: GR, TG. Supervision: AP, MM, FT. Validation: GR, AP, TG, MM, FT, MT. Visualization: GR, AP, TG, MM, FT, MT. Writing – original draft: GR, AP, TG, MM, FT, MT. Writing – review & editing: GR, AP, TG, MM, FT, MT.

## Conflict of Interest Statement

The authors declare that the research was conducted in the absence of any commercial or financial relationships that could be construed as a potential conflict of interest.

## Abbreviations

CFs, Contextual Factors; EQI, EuroQol Index; CHERRIES, Checklist for Reporting Results of Internet E-Surveys; STROBE, STrengthening the Reporting of OBservational Studies in Epidemiology.
