# Supplementary material for: The Knowledge of Contextual Factors as Triggers of Placebo and Nocebo Effects in Patients With Musculoskeletal Pain: Findings From a National Survey
Source: Front Psychiatry. 2019 Jul 4;10:478. doi: 10.3389/fpsyt.2019.00478 (PMC6620866; doi:10.3389/fpsyt.2019.00478)
Supplement: Supplementary file 1 [file Table_1.docx]

**Welcome to this survey!**

Dear patient,

Thank you for taking part in this survey. This survey aims to clarify the use of contextual factors in enhancing the therapeutic outcome in clinical practice.

Contextual factors consist in a series of relational or environmental situations that may affect your perception on patients’ suffering and functional impairment (e.g. the role of the tone of the voice used by the clinician in healing a back pain, or the role of the design of the therapeutic setting in influencing post-treatment wellbeing). Examples of the main contextual factors are: words and posture used by the clinician, smells, sounds and decor of the therapeutic setting to enhance the effectiveness of the treatment.

We consider important to study them in their different applications within the clinical practice.

Please answer the following questions based on your personal experience. Completion of the entire questionnaire is voluntary and will take you 10 to 15 minutes. Your answers are completely anonymous and will only be used for the purposes of this research.

By clicking on the link to the survey, you provide your consent to participate in the study. Whenever you complete the page, click on "Next" to save your answer. If you decide to abandon the survey, click on "Exit".

**Socio-demographic characteristics**

***What is your gender?*** *[Please select]*

- Male
- Female

***How old are you?*** *[Please complete]*

……….

***Which part of Italy do you live in?*** *[Please select]*

- North
- Center
- South

***What is your social status?*** *[Please select]*

- Student
- Employed
- Unemployed
- Retired
- Housewife

***What is your type of job?*** *[Please select]*

- Legislator, businessman, manager
- Intellectual, scientific, highly specialized profession
- Technician
- Office worker
- Trade, service
- Laborer, farmer, artisan
- Drivers
- Unqualified profession
- Military profession

***What is your highest education?*** *[Please select]*

- Primary school
- Secondary school
- High school
- Bachelor’s degree
- PhD
- Master’s degree

***Where do you currently feel pain ?*** *[Please select]*

- Lumbar spine-pelvis
- Cervical spine-head
- Thoracic spine-ribs
- Jaw
- Shoulder-arm
- Elbow-forearm
- Wrist-hand
- Hip-thigh
- Knee-leg
- Ankle-foot

***How long have you been in pain?*** *[Please select]*

- Less than 3 months
- From 3 months to 6 months
- Over 6 months

***How intense is your perceived pain, from 0 (no pain) to 10 (maximum pain)?*** *[Please select]*

- 0
- 1
- 2
- 3
- 4
- 5
- 6
- 7
- 8
- 9
- 10

**Now we are going to offer you a typical situation that reflects what many patients experience in clinician practice.**

**Clinical vignette 1**

A 40-year-old man, freelancer, visits your clinic complaining about low back pain. He requests a massage to return to work more quickly. Based on clinical examination the clinician doesn’t find contraindications for the use of massage, but you know that in this case there is no indication to use this therapy. The patient insists on requiring massage on the grounds that this therapy helped him in the past during a previous episode of low back pain.

***What should the clinician do in this situation?*** *[Please select]*

- (A) Deliver the massage
- (B) Tell the patient that low back pain would resolve itself in a few days
- (C) Suggest the possibility of delivering massage if the clinical condition fails to improve
- (D) Advise a different treatment commonly used for low back pain
- (E) Try to convince the patient of the futility of massage

**Clinical vignette 2**

In a hospital, a patient with high shoulder pain receives laser therapy on demand, several times a day. In case of frequent requests therapy is occasionally replaced with sham laser (with power-off) aimed at avoiding lesion to tissues. The patient reports that in each case laser (whether active or sham) improved their clinical condition.

***What conclusion can be drawn on the effectiveness of laser sham?*** *[More answers are possible]*

- (A) The positive attention of the clinician leads to decreased pain
- (B) Pain is not organic but psychological
- (C) The patient is very suggestible
- (D) The supporting patient determined improvements after treatment with sham laser (power-off)

**Beliefs**

Context factors can influence the perception of your condition of suffering (e.g. pain, stiffness, weakness). Examples of the main contextual factors are: the words and posture used by the clinician, the relationship between the patient and the clinician, the smells, the sounds, the light and the furnishing of the healthcare setting.

***How much do you believe that the therapeutic outcome (e.g. improvement of low back pain) can be influenced positively by...?*** *[Please select]*

|  | **Very Much** | **Much** | **Enough** | **Few** | **None** |
| --- | --- | --- | --- | --- | --- |
| Clinician’s professional reputation |  |  |  |  |  |
| Clinician’s uniform |  |  |  |  |  |
| Clinician’s positive attitudes and behavior |  |  |  |  |  |
| Patient’s expectation and preference |  |  |  |  |  |
| Patient’s previous experience |  |  |  |  |  |
| Verbal communication |  |  |  |  |  |
| Non-verbal communication |  |  |  |  |  |
| Empathetic therapeutic alliance with the patient |  |  |  |  |  |
| Overt therapy |  |  |  |  |  |
| Patient-centered approach |  |  |  |  |  |
| Professional approach to patient |  |  |  |  |  |
| Physical contact with the patient |  |  |  |  |  |
| Comfortable setting |  |  |  |  |  |
| Adequate environmental architecture |  |  |  |  |  |
| Adequate design |  |  |  |  |  |

**Ethical issues**

***The use of contextual factors for therapeutic purposes can be considered ethically acceptable when ...*** *[You can select more than one answer]*

- (A) It exerts positive psychological effects
- (B) The other therapies are over
- (C) The patient wants or expects this treatment
- (D) Their effectiveness is shown by clinical experience

***The use of contextual factors for therapeutic purposes can be considered ethically unacceptable when ...*** *[You can select more than one answer]*

- (A) It is based on deception
- (B) It undermines trust between patient and clinician
- (C) Evidence is insufficient

**Communication and application**

***How would you receive communication from a clinician about the use of contextual factors?*** *[You can select multiple possibilities]*

- (A) It is a treatment that can help and will not hurt
- (B) It is an effective treatment
- (C) It is a treatment without a specific effect for your problem, but capable of improving your condition
- (D) It is a treatment that induces psychological change
- (E) It can help but you are not sure about its effect
- (F) You do not receive any information

***Under what circumstances should a clinician adopt contextual factors?*** *[You can select multiple possibilities]*

- (A) As a result of unjustified and constant demands for healthcare interventions
- (B) To calm down the patient
- (C) When all other therapies are over
- (D) In addition to other interventions to optimize clinical responses
- (E) For non-specific problems
- (F) To gain time
- (G) As a diagnostic tool to differentiate between psychological and physiological problems
- (H) To control pain

**Mechanism of action, therapeutic effect and definition**

***What mechanisms of action can explain the effect of contextual factors?*** *[You can select multiple possibilities]*

- (A) Patient’s expectations
- (B) Conditioning
- (C) Suggestibility
- (D) Natural history of the disease
- (E) Psychological factors
- (F) Unexplained
- (G) Physiological/biological factors
- (H) Spiritual energies
- (I) Mind-body connections

***What are, in your opinion, the potential effects of contextual factors in the following health problems?*** *[Please select]*

|  | **Psychological** | **Physiological** | **Psychological and Physiological** | **No benefit** |
| --- | --- | --- | --- | --- |
| Acute pain |  |  |  |  |
| Chronic pain |  |  |  |  |
| Cognitive disorder |  |  |  |  |
| Emotional disorder |  |  |  |  |
| Gastrointestinal disorder |  |  |  |  |
| Sexual disorder |  |  |  |  |
| Drug and medication addiction |  |  |  |  |
| Neurological disorder |  |  |  |  |
| Rheumatologic disorder |  |  |  |  |
| Immune/allergic disorder |  |  |  |  |
| Oncological disorder |  |  |  |  |
| Cardiovascular disorder |  |  |  |  |
| Infections |  |  |  |  |
| Insomnia |  |  |  |  |

***How would you define, in the light of this survey, the therapeutic role of contextual factors?*** *[Please select]*

- (A) An intervention without a specific effect for the condition being treated, but with a possible aspecific effect’
- (B) An intervention that has a special effect through known physiological mechanisms
- (C) A harmless or inert intervention
- (D) A sham treatment used as control tests for the safety and efficacy of the active treatment

***Dear patient, thanks for taking the time to complete this survey!***
