# Supplementary material for: The Knowledge of Contextual Factors as Triggers of Placebo and Nocebo Effects in Patients With Musculoskeletal Pain: Findings From a National Survey
Source: Front Psychiatry. 2019 Jul 4;10:478. doi: 10.3389/fpsyt.2019.00478 (PMC6620866; doi:10.3389/fpsyt.2019.00478)
Supplement: Supplementary file 2 [file Table_2.docx]

**Benvenuto a questa indagine**

Gentile signora/e grazie per prendere parte a questo questionario.

Questa indagine ha l’intento di raccogliere o esplorare la percezione sull’utilizzo dei *fattori di contesto* per il potenziamento del risultato terapeutico nell’attività clinica.

I fattori di contesto sono costituiti da una serie di situazioni relazionali o ambientali che possono influenzare la percezione che Lei ha della sua condizione di sofferenza e di limitazione funzionale (per esempio il ruolo che ha il tono della voce usato dal clinico nella guarigione di un mal di schiena, quanto incide l’arredamento dello studio nel benessere post trattamento, …). I principali fattori di contesto sono le parole e la postura utilizzata dal clinico, gli odori, i suoni e l’arredamento del setting terapeutico. Noi consideriamo importante studiarli nella pratica clinica quotidiana.

Gentilmente risponda alle seguenti domande sulla base della Sua personale esperienza. La compilazione dell’intero questionario è volontaria e richiede 10-15 minuti. Le Sue risposte sono completamente anonime e saranno utilizzate solamente per gli scopi di questa ricerca.

Cliccando sul link del questionario, Lei fornisci il tuo consenso a partecipare allo studio. Quando completa la pagina, clicchi su “Prosegui” per salvare le tue risposte. Se decide di abbandonare il questionario, selezioni “Uscita”.

**Caratteristiche socio-demografiche**

***Quale è il suo sesso?*** *[Selezioni]*

- Maschio
- Femmina

***Quanti anni ha?*** *[Completi numericamente es. 32]*

……….

***In che regione dell’Italia lavori?*** *[Selezioni]*

- Nord
- Centro
- Sud

***Quale è il suo status sociale attuale?*** *[Selezioni]*

- Studentessa/studente
- Lavoratrice/lavoratore
- Disoccupata/disoccupato
- Pensionata/pensionato
- Casalinga/casalingo

***Quale è il suo ambito lavorativo attuale?*** *[Selezioni]*

- Legislatori, imprenditori e alta dirigenza
- Professioni intellettuali, scientifiche e di elevata specializzazione
- Professioni tecniche
- Professioni esecutive nel lavoro d’ufficio
- Professioni qualificate nelle attività commerciali e nei servizi
- Artigiani, operai specializzati e agricoltori
- Conduttori di impianti, operai di macchinari fissi e mobili e conducenti di veicoli
- Professioni non qualificate
- Forze armate

***Quale è il titolo di studio più elevato che ha raggiunto?*** *[Selezioni]*

- Scuola elementare
- Scuola media
- Scuola superiore
- Laurea
- Dottorato di ricerca
- Master/corso di formazione avanzata

***In quale di queste zone corporee percepisce dolore attualmente?*** *[Selezioni]*

- Lombare-bacino
- Cervicale-testa
- Dorsale-coste
- Mandibola
- Spalla-braccio
- Gomito-avambraccio
- Polso-avambraccio
- Anca-coscia
- Ginocchio-gamba
- Caviglia-piede

***Da quanto tempo percepisce dolore?*** *[Selezioni]*

- Da meno di 3 mesi
- Da 3 a 6 mesi
- Oltre i 6 mesi

***Quanto è intenso il dolore che percepisce, da 0 (dolore assente) a 10 (dolore massimo)?*** *[Selezioni]*

- 0
- 1
- 2
- 3
- 4
- 5
- 6
- 7
- 8
- 9
- 10

**Le proponiamo ora una situazione tipica, che riflette quanto provano molti pazienti che giungono alla attenzione del clinico.**

**Scenario clinico 1**

Un ragazzo di 40 anni libero professionista lamenta mal di schiena e vorrebbe essere trattato con massaggio per poter tornare a lavorare prima. Il clinico non riscontra problemi per l’utilizzo del massaggio, ma sa che in questo caso non è la terapia indicata e il mal di schiena si risolverebbe spontaneamente in qualche tempo. Il paziente insiste in modo convinto a richiedere di essere trattato con massaggio, basandosi sul fatto che questo lo ha aiutato in passato durante un precedente episodio di lombalgia.

***Secondo lei cosa dovrebbe fare il clinico in questa situazione?*** *[Selezioni]*

- (A) Erogare il massaggio
- (B) Poiché è probabile che quel tipo di mal di schiena guarisca da solo in qualche tempo, dovrebbe spiegarlo al paziente e non erogare il massaggio
- (C) Proporre la possibilità di erogare il massaggio successivamente qualora il mal di schiena non migliori
- (D) Proporre un trattamento diverso comunemente utilizzato per il mal di schiena
- (E) Provare a convincere il paziente che non necessita di massaggio

**Scenario clinico 2**

In un ospedale, un paziente con un forte dolore alla spalla riceve un trattamento con il laser più volte al giorno su sua richiesta. Per le frequenti richieste, occasionalmente la terapia con il laser viene eseguita con apparecchio spento all’insaputa del paziente (per evitare danni ai tessuti, compiacendo comunque il paziente). Il paziente riporta che ha avuto buoni risultati anche quando l’apparecchio era spento.

***Che conclusione può trarre circa l’efficacia del laser effettuato con apparecchio spento?*** *[Può selezionare più di una risposta]*

- (A) Il clinico si prende cura del paziente, che sta meglio perché riceve attenzione
- (B) Il dolore non aveva cause fisiche, ma psicologiche
- (C) Il paziente era molto suggestionabile
- (D) Assecondare il paziente ha determinato i miglioramenti dopo il trattamento con il laser spento

**Credenze**

I fattori di contesto possono influenzare la percezione che Lei ha della sua condizione di sofferenza (es. dolore, rigidità, debolezza). Ne sono esempi: le parole e la postura utilizzata dal clinico, il rapporto tra paziente e clinico, gli odori, i suoni, la luce e l’arredamento dell’ambulatorio.

***Quanto CREDE che il risultato terapeutico (es. il miglioramento di un mal di schiena) possa essere influenzato positivamente dal fatto che il clinico…?*** *[Selezioni]*

|  | **Moltissimo** | **Molto** | **Abbastanza** | **Poco** | **Per nulla** |
| --- | --- | --- | --- | --- | --- |
| Parli della propria esperienza, del proprio percorso professionale |  |  |  |  |  |
| Indossi una uniforme che lo identifichi come clinico |  |  |  |  |  |
| Adotti atteggiamenti e comportamenti ottimistici verso il suo stato di salute |  |  |  |  |  |
| Si dimostri disponibile ad assecondare le sue richieste e preferenze di trattamento (es. massaggio, esercizio, etc.) |  |  |  |  |  |
| Per la scelta del trattamento tenga conto delle sue precedenti esperienze, sia positive che negative |  |  |  |  |  |
| Utilizzi un linguaggio semplice e comprensibile |  |  |  |  |  |
| Utilizzi postura, gesti, espressioni facciali, sorriso mentre le comunica il problema per metterla a suo agio |  |  |  |  |  |
| Stabilisca con lei una buona relazione dal punto di vista umano |  |  |  |  |  |
| La informi sulle caratteristiche della terapia che sta effettuando (controindicazioni, tempi di recupero, perché è stata scelta, etc.) |  |  |  |  |  |
| Utilizzi un approccio terapeutico individualizzato (tenga conto delle sue specifiche esigenze quali il contesto lavorativo, sociale etc.) |  |  |  |  |  |
| Rispetti la puntualità e sia rispettoso della sua privacy |  |  |  |  |  |
| Appoggi la sua mano sulla spalla per rassicurarla o mentre la informa sulla cura |  |  |  |  |  |
| Eserciti in un ambiente confortevole (es. poco rumoroso, con musica, profumi e temperatura adeguata, etc.) |  |  |  |  |  |
| Eserciti in un ambiente con architettura adeguata dell’ambiente (es. con finestre e punti luce, indicazioni e segnaletica chiara, etc.) |  |  |  |  |  |
| Eserciti in un ambiente dal design ambientale accurato (es. con decorazioni, quadri, etc.) |  |  |  |  |  |

**Eticità**

***Secondo Lei l’utilizzo dei fattori di contesto a fini terapeutici può essere considerato eticamente accettabile (quindi corretto e non un inganno per il paziente) quando...*** *[Può selezionare più di una risposta]*

- (A) Induce effetti psicologici benefici
- (B) Tutte le altre terapie sono terminate e si vuole tentare un'altra strada
- (C) La paziente/il paziente vuole o si aspetta quel trattamento
- (D) Il clinico ha già provato con altri pazienti con risultato positivo

***Secondo Lei l’utilizzo dei fattori di contesto a fini terapeutici può essere considerato eticamente non accettabile (quindi scorretto) quando****... [Può selezionare più di una risposta]*

- (A) E' basato sull'inganno
- (B) Può rovinare la fiducia tra clinico e paziente
- (C) Le prove scientifiche non sono sufficienti a dire che i fattori contesto sono utili per quel tipo di paziente

**Comunicazione ed applicazione**

***Come vorrebbe le fosse comunicato l’utilizzo dei fattori di contesto dal clinico?*** [*Può selezionare più di una risposta]*

- (A) E' un trattamento che può aiutare e non farà male
- (B )E' un trattamento efficace
- (C) E' un trattamento che pur non agendo specificamente sul suo problema genera un miglioramento della sua condizione (ad es. non cambia la forza muscolare, potrebbe ridurre la percezione del dolore)
- (D) E' un trattamento che induce miglioramenti psicologici
- (E) E' un trattamento che può aiutare, anche se non si sa esattamente come agisca
- (D) Non ricevere nessuna informazione

***In che circostanze ritiene sia utile/corretto che il clinico sfrutti i fattori di contesto?*** *[Può selezionare più di una risposta]*

- (A) Per soddisfare la richiesta del paziente di ricevere trattamenti sanitari non giustificabili dalla condizione clinica
- (B) Per calmare il paziente (es. usando una luce più bassa può ridurre l’ansia)
- (C) Dopo che tutte le altre terapie non hanno avuto l’effetto sperato
- (D) Come aggiunta agli altri interventi sanitari per ottimizzare il risultato
- (E) Per problemi non specifici (es. mal di schiena persistente in assenza di fratture o infezioni, …)
- (F) Per guadagnare tempo in attesa che il corpo guarisca spontaneamente
- (G) Come strumento per diagnosticare se il dolore è di tipo psicologico o fisico
- (H) Per controllare il dolore

**Meccanismo di azione, effetto terapeutico e definizione**

***Come si spiega, secondo lei, l’effetto dei fattori di contesto?*** *[Può selezionare più di una risposta]*

- (A) Le aspettative del paziente (gli è stato dato quello che voleva)
- (B) Il condizionamento (l’amico che ha parlato al paziente del clinico l’ha convinta che è bravo)
- (C) La suggestionabilità del paziente
- (D) La storia naturale del disturbo (sarebbe guarito comunque)
- (E) I fattori psicologici (il paziente è stato influenzato psicologicamente a stare meglio)
- (F) Non spiegabile
- (G) Fattori fisiologici/biologici (si è verificato un miglioramento fisico dei tessuti danneggiati)
- (H) Energie spirituali positive
- (I) Connessione mente-corpo (se la mente è a proprio agio, anche il corpo migliora)

***Quali sono, secondo lei, i MIGLIORAMENTI indotti dai fattori di contesto (… ) nei seguenti problemi di salute?*** *[Selezioni]*

|  | **Benessere mentale** | **Benessere fisico** | **Benessere mentale e fisico** | **Nessun beneficio** |
| --- | --- | --- | --- | --- |
| Dolore acuto (es. mal di schiena da 3 giorni) |  |  |  |  |
| Dolore cronico (es. mal di schiena da 1 anno) |  |  |  |  |
| Problemi cognitivi (es. demenza) |  |  |  |  |
| Problemi emozionali (es. ansia, depressione …) |  |  |  |  |
| Problemi gastrointestinali (es. reflusso, gastrite, colite…) |  |  |  |  |
| Problemi sessuali (impotenza, alterazioni del ciclo, …) |  |  |  |  |
| Dipendenze da droghe o farmaci |  |  |  |  |
| Problemi neurologici (es. ictus, Parkinson …) |  |  |  |  |
| Problemi reumatologici (es. artrosi, osteoporosi, …) |  |  |  |  |
| Problemi immunitari/allergie (es. allergie stagionali, …) |  |  |  |  |
| Problemi oncologici (es. cancro…) |  |  |  |  |
| Problemi cardiovascolari (es. infarto, aritmia, …) |  |  |  |  |
| Infezioni |  |  |  |  |
| Insonnia |  |  |  |  |

***Come DEFINIREBBE, alla luce di questa indagine il ruolo terapeutico dei fattori di contesto?*** *[Selezioni]*

- (A) Un intervento che non cura specificamente il vero problema del paziente, ma può influenzare il risultato clinico
- (B) Un intervento che cura il vero problema del paziente
- (C) Un intervento che non ha alcun effetto sui risultati clinici, ma solo sulla qualità percepita dal paziente del trattamento ricevuto
- (D) Un intervento placebo usato come test di controllo per la sicurezza e l’efficacia di un trattamento attivo (es. confronto il placebo col massaggio per vedere se funziona davvero o se il paziente guarisce perché suggestionabile)

**Caro paziente grazie per aver preso parte a questa indagine!**
